# Virus-Like Particles of Chimeric Recombinant Porcine Circovirus Type 2 as Antigen Vehicle Carrying Foreign Epitopes

**DOI:** 10.3390/v6124839

**Published:** 2014-12-05

**Authors:** Huawei Zhang, Ping Qian, Lifeng Liu, Suhong Qian, Huanchun Chen, Xiangmin Li

**Affiliations:** 1 State Key Laboratory of Agricultural Microbiology, Huazhong Agricultural University, Wuhan 430070, Hubei, China; E-Mails: azhangzhizhong@126.com (H.Z.); qianp@mail.hzau.edu.cn (P.Q.); liu.lifeng@outlook.com (L.L.); qiansuhong@yeah.net (S.Q.); chenhch@mail.hzau.edu.cn (H.C.); 2 Laboratory of Animal Virology, College of Veterinary Medicine, Huazhong Agricultural University, Wuhan 430070, Hubei, China

**Keywords:** porcine circovirus type 2, capsid protein, T-cell epitope, B-cell epitope, virus-like particle, immunogenicity

## Abstract

Virus-like particles (VLPs) of chimeric porcine circovirus type 2 (PCV2) were generated by replacing the nuclear localization signal (NLS; at 1–39 aa) of PCV2 capsid protein (Cap) with classical swine fever virus (CSFV) T-cell epitope (1446–1460 aa), CSFV B-cell epitope (693–716 aa) and CSFV T-cell epitope conjugated with B-cell epitope. The recombinant proteins were expressed using the baculovirus expression system and detected by immunoblotting and indirect immunofluorescence assay. The abilities to form PCV2 VLPs were confirmed by transmission electron microscopy. Immunogenicities of the three recombinant proteins were evaluated in mice. Our Results indicated that Cap protein NLS deletion or substitution with CSFV epitopes did not affect the VLPs assembly. Three chimeric Cap proteins could form VLPs and induce efficient humoral and cellular immunity against PCV2 and CSFV in mice. Results show that PCV2 VLPs can be used as an efficient antigen carrier for delivery of foreign epitopes, and a potential novel vaccine.

## 1. Introduction

Classical swine fever (CSF) caused by CSF virus (CSFV) is a highly contagious and devastating disease of swine worldwide [1,2]. CSFV belongs to the *Pestivirus* genus of the *Flaviviridae* family [3,4]. The viral genome of CSFV is a single- and positive-stranded RNA of approximately 12.3 kb, which contains a large open reading frame encoding a polyprotein of 3898 amino acids [1,5]. The viral protein includes four structural (C, Erns, E1 and E2) and eight non-structural proteins (Npro, P7, NS2, NS3, NS4A, NS4B, NS5A and NS5B) [6,7].

The E2 envelope glycoprotein is the key immunogenic protein that elicits protective immunity against CSFV infection in pigs. This protein contains sequential neutralizing epitopes and has been used as the main component in the design of CSFV–DIVA vaccines [7]. The amino acid sequence (CKEDYRYAISSTNEIGLLGAGGLT) of the E2 glycoprotein (693–716 aa) is one of the major epitopes with neutralizing activity [8,9,10,11]. Previous studies suggested that this major epitopes can completely or partially protect pigs against CSFV [11,12]. The amino acid residues (1446–1460) of the non-structural protein NS3 (KHKVRNEVMVHWFDD) contains a specific helper T-cell epitope and a CTL epitope and play important roles in humoral and cellular immunity [8,9,12].

Porcine circovirus type 2 (PCV2), an important porcine pathogen, primarily causing PCV2-associated diseases in pigs [13,14]. The genome of PCV2 is a small single-stranded circular DNA with 1.76-kb length, which encodes replicase, capsid (Cap) protein and other viral proteins. PCV2 belongs to the *Circovirus* genus of the *Circoviridae* [15]. The Cap protein of PCV2 can be independently assembled into virus-like particles (VLPs); the Cap protein contains a nuclear localization signal (NLS) at its *N*-terminus [16]. Studies have shown that recombinant Cap proteins containing NLS sequence or not could assemble into VLPs in the eukaryotic and prokaryotic expression systems [16,17,18]. The Cap protein is a major immunogenic protein and an important target antigen for developing novel vaccines against PCV2 infection [19,20].

Generally, VLPs are non-infectious and can effectively stimulate humoral and cell-mediated immune responses [20,21]. Moreover, VLPs can be easily produced on a large scale [22]. Therefore, VLPs are ideal approaches for the design of foreign epitope carriers and gene transfer vehicles. Currently, many animal virus VLPs have been used as delivery systems for antigenic epitopes to induce immune responses against target antigen [23]. For example, the VLPs formed by porcine parvovirus (PPV) VP2 have been successfully used as foreign epitope carriers. As a foreign gene, the CD8^+^ T-cell epitope from the lymphocytic choriomeningitis virus (LCMV) can be inserted into the *N*-terminus of VP2 without altering the assembly of VLPs. Immunization with the chimeric VLPs of PPV in mice can elicit strong CTL responses and confer total protection against the lethal LCMV challenge [24].

In this study, we evaluated the potential of PCV2 VLPs as a vessel for carrying foreign epitopes. The CSFV T-cell epitope (1446–1460 aa), CSFV B-cell epitope (693–716 aa) and CSFV T-cell epitope conjugated with B-cell epitope were used as foreign epitopes to replace the NLS sequence at the *N*-terminus of the PCV2 Cap protein. Three recombinant proteins were then expressed in the baculovirus system. Their immunogenicity were subsequently valuated in mice. All of them induced well humoral and cellular immunity. To our knowledge, this study is reported for the first time.

## 2. Materials and Methods

### 2.1. Viruses, Cells and Plasmids

The recombinant baculovirus (Ac-Cap) was previously constructed in our laboratory. PK-15 cells, free of PCV1 and PCV2, were cultured in Dulbecco’s Minimal Essential Medium (DMEM; Gibco, Grand Island, NY, USA) with 10% fetal bovine serum (FBS; Gibco, Grand Island, NY, USA) at 37 °C in 5% CO_2_. Sf9 cells were propagated at 27 °C in Grace’s medium (Invitrogen, Carlsbad, CA, USA) supplemented with 10% FBS. PCV2 WH strain was provided by Professor Qigai He (Huazhong Agricultural University, Wuhan, China). 50% confluent PK-15 cells in T-25 flasks were inoculated with PCV2 WH strain virus at a multiplicity of infection (MOI) of 1. After 1 h at 37 °C, the supernatant was removed and washed twice with DMEM, and the cells were maintained in DMEM with 1% fetal bovine serum. Then the virus was collected at 84 h post-inoculation (hpi).

**Figure 1:**
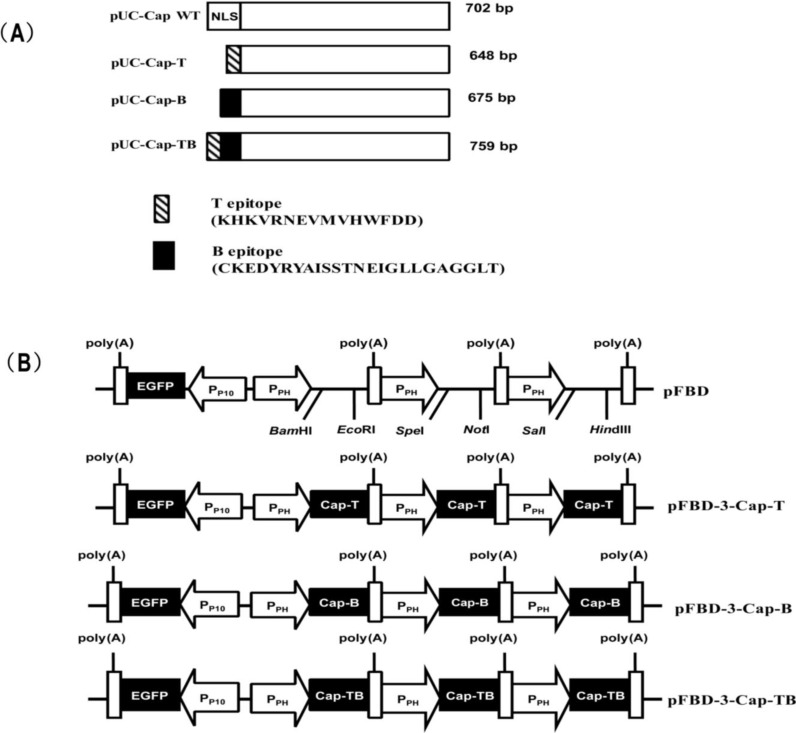
Schematic diagrams of the recombinant transfer vector (pFBD, pFBD-3-Cap-T, pFBD-3-Cap-B and pFBD-3-Cap-TB). (**A**) Schemes of chimeric Cap protein genes. NLS, the nuclear localization signal of Cap protein; the black boxes, the CSFV B-cell epitope (693–716 aa); the twill boxes, the CSFV T-cell epitope (1446–1460 aa); the white boxes, the gene of Cap; (**B**) Scheme of each recombinant transfer vector. The eGFP expression cassette was driven by the p10 promoter (P_P10_); P_PH_, the polyhedrin promoter of baculovirus; poly(A), polyadenylation signal.

The CSFV T-cell epitope (1446–1460 aa), CSFV B-cell epitope (693–716 aa) and CSFV T-cell epitope conjugated with B-cell epitope were synthesized (Sangon Biotech, Shanghai, China) for replacing the NLS amino acid of the Cap protein genes (1–39 aa). These epitopes were cloned into the pUC59 vector and designated as pUC-Cap-T, pUC-Cap-B and pUC-Cap-TB, respectively (Figure 1A). Plasmid pFastBac Dual™ (Invitrogen) was used as the backbone of the baculovirus transfer vector and named as pFBD (Figure 1B). The pFBD transfer vector, which expresses three genes by separate polyhedron promoters, was previously constructed in our laboratory according to the method of Pushko [25]. First, a *Stu*I-*EcoR*I fragment of chicken β-globin polyadenylation signal from plasmid pCAGGS was inserted in corresponding sites, and driven by the AcMNPV polyhedrin promoter (P_PH_) within pFastBac Dual™ transfer vector to construct plasmid pFBD-gpA. The gene of eGFP was cloned and inserted into pFBD-gpA downstream of the AcMNPV p10 promoter (P_P10_) to gain pFBD-gpA-P10eGFP. Then a *Xho*I-*Hind*III fragment containing the sequence of P_PH_ flanked with *Spe*I and *Not*I restriction site, SV40 polyadenylation signal, another sequence of P_PH_ with *Xho*I and *Hind*III restriction site, were synthesized (Sangon Biotech, Shanghai, China) and inserted into pFBD-gpA-P10eGFP digested with *Xho*I and *Hind*III to gain transfer vector pFBD.

### 2.2. Construction of Recombinant Baculoviruses

All primers used in this study are summarized in Table 1. To obtain the recombinant construct pFBD-3-Cap-T, we performed three separate PCR reactions using the plasmid pUC-Cap-T as the template. The three PCR products were inserted into the pFBD vector. Finally, the transfer vector pFBD-3-Cap-T encoding three copies of Cap-T was obtained; each Cap-T copy was obtained within its own expression cassette, including a polyhedrin promoter and a transcription termination sequence (Figure 1B). Another two transfer vectors (namely pFBD-3-Cap-B and pFBD-3-Cap-TB) expressed three copies of the Cap-B and Cap-TB genes, respectively, were constructed using the same method (Figure 1B). All recombinant transfer vectors were confirmed through sequencing. Three recombinant baculoviruses, namely, Ac-Cap-T, Ac-Cap-B and Ac-Cap-TB, were subsequently generated using the Bac-to-Bac system (Invitrogen) following the manufacturer’s instructions.

**Table 1:** Primers used in this study.

Genes	Name	Primer sequence (5'–3')	Restriction site
Cap-T ^a^	1-F	TT**GGATCC**GCCACCATGAAACACAAAGTGAGGAATGAAGT	*Bam*HI
	2-F	TT**ACTAGT**GCCACCATGAAACACAAAGTGAGGAATGAAGT	*Spe*I
	3-F	TT**GTCGAC**GCCACCATGAAACACAAAGTGAGGAATGAAGT	*Sal*I
Cap-B ^b^	4-F	TT**GGATCC**GCCACCATGTGCAAGGAAGATTACAGGT	*Bam*HI
	5-F	TT**ACTAGT**GCCACCATGTGCAAGGAAGATTACAGGT	*Spe*I
	6-F	TT**GTCGAC**GCCACCATGTGCAAGGAAGATTACAGGT	*Sal*I
Cap ^c^	1-R	TT**GAATTC**TTACTTTGGGTTCAGTGGAGGGTCCT	*Eco*RI
	2-R	TT**GCGGCCGC**TTACTTTGGGTTCAGTGGAGGGTCCT	*Not*I
	3-R	TT**AAGCTT**TTACTTTGGGTTCAGTGGAGGGTCCT	*Hind*III

^a^ Primers used to the clone the Cap-T and Cap-TB gene’s upstream; ^b^ Primers used for the clone of the Cap-B gene’s upstream; ^c^ Primers used for clone of the Cap-T, Cap-B and Cap-TB gene’s downstream. The sequences in bold represent the corresponding restriction site. The underlined sequence indicates the kozak sequence.

### 2.3. Western Blot

The Sf9 cells were seeded at a concentration of 1.5 × 10^5^ cells/well into 6-well tissue culture plates and were infected with the four recombinant baculoviruses (Ac-Cap, Ac-Cap-T, Ac-Cap-B and Ac-Cap-TB) at a multiplicity of infection (MOI) of 1. The cells were incubated for 2 h at 27 °C. After removal of virus, fresh medium was added and incubated at 27 °C. After 72 h post-infection, the infected cell lysates were subjected to 12% sodium dodecyl sulfate–polyacrylamide gel electrophoresis and transferred onto polyvinylidene fluoride membranes. The membrane was blocked using TBS–5% (*w*/*v*) skim milk and incubated for 2 h at room temperature with anti-Cap MAb (1:100 dilution). The antibodies of anti-Cap were generated in our laboratory. After washing for three times with TBS–0.05% Tween 20, the membranes were incubated for 2 h at room temperature with horseradish peroxidase-conjugated goat anti-mouse IgG (1:3000 dilution, ABclonal, Wuhan, Hubei, China). The membranes were subsequently washed with TBS–0.05% Tween 20 for three times. Specific protein bands were visualized with ECL chemiluminescence system using an Image Lab software 4.0.1 (Bio-Rad, Shanghai, China, 2012).

### 2.4. Confocal Microscopy

The Sf9 cells cultured in 12-well plates were infected with the four recombinant baculoviruses (Ac-Cap, Ac-Cap-T, Ac-Cap-B and Ac-Cap-TB) at an MOI of 1. After 48 h post-infection, the cells were fixed using cold methanol/acetone (1:1) for 30 min at −20 °C and then blocked with 2% bovine serum albumin for 1 h at room temperature. The cells were incubated with anti-Cap MAb (1:100 dilution) for 1 h at 37 °C and then washed with PBS for three times. Subsequently, the cells were incubated with fluorescein isothiocyanate (FITC)-labeled-goat anti-mouse IgG (1:100 dilution, ABclonal) for 1 h at 37 °C and then washed with PBS for three times. The cells were analyzed using a confocal microscope (LSM 510, Carl Zeiss, Heidenheim, Germany).

### 2.5. Electron Microscopy

Sf9 cell monolayers were infected with the recombinant baculoviruses at an MOI of 1. After incubation for 4–5 days, the infected cells were washed with PBS and lysed through freezing and thawing for three times. The supernatant was centrifuged at 8000 rpm for 10 min at 4 °C. Subsequently, the supernatant was centrifuged at 30,000 rpm for 6 h with a Beckman SW28 rotor using a 40% sucrose cushion. The pellets were resuspended in 1× PBS. Protein samples were harvested for electron microscopy analysis using an H-7000FA electron microscope (Hitachi Co., Tokyo, Japan).

### 2.6. Immunization of Mice

Animal experiments were performed according to the protocols approved by the Biological Studies Animal Care and Use Committee in Hubei Province, China. The experimental design is shown in Table 2. Six-week-old female BALB/c mice were purchased from the Hubei Centre of Disease Control, Wuhan, Hubei, China. The mice were SPF grade (specific pathogen-free) and were randomly divided into four groups of six mice. Groups A, B and C were intramuscularly vaccinated with 200 μL of the solution containing 40 µg of recombinant proteins (Cap-T, Cap-B or Cap-TB) with ISA-206 adjuvant (Seppic, Paris, France) at a ratio of 50:50 (*w*/*w*) according to the manufacturer’s instructions. Group D, the negative control group, were immunized with 200 µL of PBS. All mice were boosted with the same dose at 14 days post-primary immunization (dpi). Blood samples for serum preparation were collected at 0, 14, 28 and 40 dpi. Serum samples were tested for PCV2-specific antibodies or CSFV-specific antibodies by indirect ELISA. The Serum samples that were collected at 14, 40 dpi as described above were analyzed for the presence of PCV2-specific neutralizing antibodies.

**Table 2:** Experimental design.

Groups	Number of mice	Recombinant proteins	Type and composition	Immunization	Number of inoculation	Concentration
A	6	Cap-T	W/O/W, ISA 206 VG	IM	2	0.2 µg/µL
B	6	Cap-B	W/O/W, ISA 206 VG	IM	2	0.2 µg/µL
C	6	Cap-TB	W/O/W, ISA 206 VG	IM	2	0.2 µg/µL
D	6	PBS	PBS	IM	2	-

IM: intramuscularly; W/O/W: water-in-oil-in-water emulsion; Adjuvant: Montanide ISA 206 VG (Seppic, France); Recombinant protein was administered twice (0, 14 dpi, days post-primary immunization).

### 2.7. Indirect ELISA

Sera was tested through indirect ELISA to assess specific antibody. Ninety-six-well flat-bottomed plates were coated with 0.1 µg of the recombinant Cap protein without NLS (the 39 *N*-terminus amino acid residues) and 2 µg of T peptides or 2 µg of B peptides diluted in 0.1 M carbonate/bicarbonate buffer (pH 9.6). After washing for three times with PBS–0.05% Tween 20, the plates were blocked with 2% BSA–PBST and then incubated with the serum samples (1:100 dilution) for 1 h at 37 °C. After washing for three times with PBS–0.05% Tween 20, we added HRP-conjugated goat anti-mouse IgG (1/5000), IgG1 (1/10,000) and IgG2a (1/5000) (ABclonal) for 1 h at 37 °C. HRP signal was detected using the tetramethylbenzidine substrate. The reaction was terminated by adding 50 µL of 2 M H_2_SO_4_. OD value was determined at 450 nm.

### 2.8. Virus Neutralization Assay

The serum samples from each group were heat inactivated at 56 °C for 30 min. 50 µL of serum two-fold serial dilutions was incubated with 50 µL of 200 TCID_50_ of the virus for 1 h at 37 °C. The serum-virus mixtures were inoculated to the confluent PK-15 cells cultured in 96-well plates and incubated at 37 °C for 3 day. Then, the cells were fixed with 1:1 acetone/methanol solution at −20 °C for 30 min and blocked with 2% BSA in PBS for 1 h at room temperature. The cells were incubated with anti-Cap MAb as the primary antibody and with a FITC-conjugated goat anti-mouse IgG (1:100 dilution, ABclonal) as the second antibody. The cells were observed using a fluorescence microscope (Olympus, Tokyo, Japan). The neutralizing antibody titers were evaluated as the reciprocal of the highest dilution that completely blocks PCV2-infection in PK-15 cells. The neutralizing neutralization assay was performed as previously described [26].

### 2.9. IFN-γ Release Assays

At 40 dpi, the presence of cytokine IFN-γ in the serum samples from vaccinated mice was detected using commercial mouse IFN-γ ELISA kits (BOSTER, Wuhan, China) according to the manufacturer’s instructions. Standard curves were generated using the control IFN-γ, which was serially diluted by twofold in PBS. The levels of serum IFN-γ were determined according to the standard curve.

### 2.10. Cytotoxicity Assay

To determine whether immunization with VLPs could induce strong CTL responses, we performed LDH release cytotoxic assay according to the manufacturer’s instructions. At 40 dpi, the splenocytes from immunized mice were suspended in a complete RPMI-1640 medium containing 10% FCS. Spleen cells were stimulated *in vitro* with CSFV T-cell epitope KHKVRNEVMVHWFDD (25 µg/mL) and recombinant human IL-2 (100 μg/mL). After 5 days post-stimulation, the splenocytes were used as effector cells in CTL assays. Target cells (p815) were also stimulated with T-cell epitope (25 µg/mL). The assays were performed in triplicate with 1 × 10^5^ targets/well at effector cell/target cell (E:T) ratios of 100:1 and 50:1. After 4-h incubation at 37 °C, the absorbance of the culture supernatant from each well was quantitatively measured using a multi-channel spectrophotometer (Bio-Rad, Hercules, CA, USA) according to the manufacturer’s instructions. The percentage of specific lysis was calculated as follows: (experimental − spontaneous release)/(maximum − spontaneous release) × 100.

### 2.11. Statistical Analysis

All data were analyzed using one-way ANOVA by GraphPad Prism software (GraphPad Prism Version 5, GraphPad Software, La Jolla, CA, USA, 2012). *p* < 0.05 were considered statistically significant.

## 3. Results

### 3.1. Expression of Recombinant Proteins

**Figure 2:**
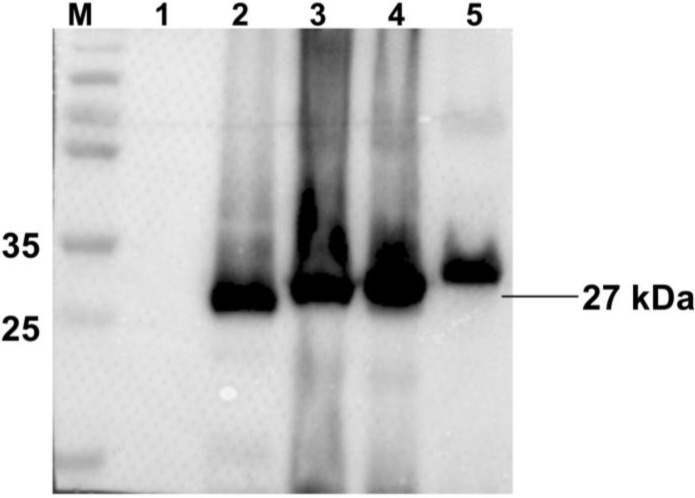
Western-blot analysis of recombinant proteins expression in Sf9 cells. Lane 1: cell lysates of normal Sf9 cells as negative control; Lane 2: cell lysates of Ac-Cap; Lane 3: cell lysates of Ac-Cap-T; Lane 4: cell lysates of Ac-Cap-B; Lane 5: cell lysates of Ac-Cap-TB. Primary antibody is the mouse anti-cap MAb and secondary antibody is the goat anti-mouse IgG-HRP.

As shown in Figure 2, no specific bands were detected in the normal Sf9 cells, whereas all recombinant proteins had a specific protein band with a molecular mass of approximately 27 kDa. All recombinant proteins had specific positive reactions with anti-Cap MAb and were successfully expressed.

**Figure 3:**
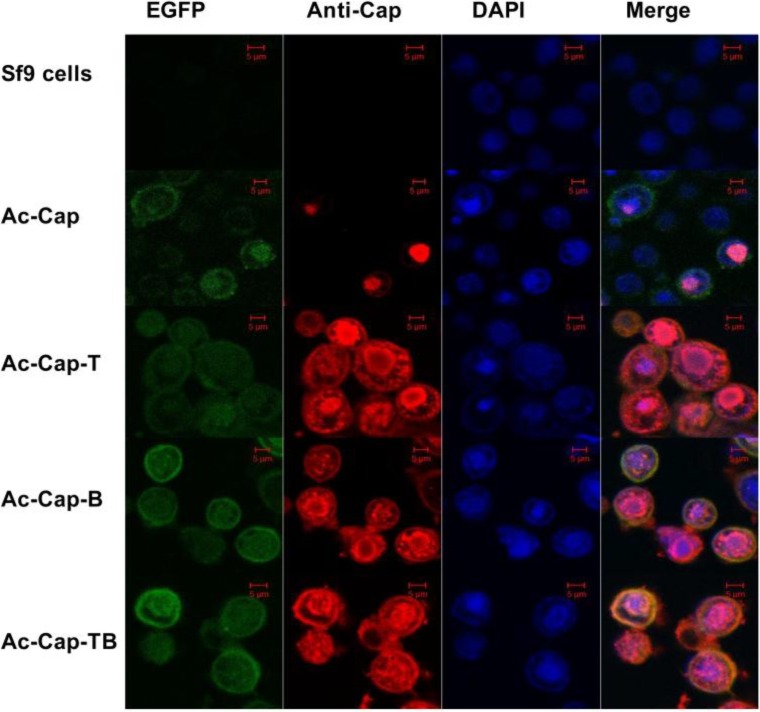
Confocal microscopy analysis of recombinant proteins expression in Sf9 cells. Sf9 cells were infected with recombinant viruses (Ac-Cap, Ac-Cap-T, Ac-Cap-B and Ac-Cap-TB), respectively. At 48 h post-infection, cells were fixed by methanol/acetone (1:1) and were analyzed by IFA using the anti-cap MAb as primary antibody and CY3-conjugated goat anti-mouse IgG as secondary antibody. Scale bar indicates 5µm.

An indirect immunofluorescence assay (IFA) was used to confirm the expression of the separate Cap recombinant proteins. The cells infected with the recombinant baculoviruses demonstrated a Cap-specific red fluorescence (Figure 3), whereas no specific red fluorescence was observed in the normal Sf9 cells (negative control). Moreover, the recombinant proteins were found in the cell nucleus as shown in Figure 3. Thus, the results indicated that all recombinant proteins were successfully expressed.

### 3.2. Electron Microscopy Analysis

**Figure 4:**
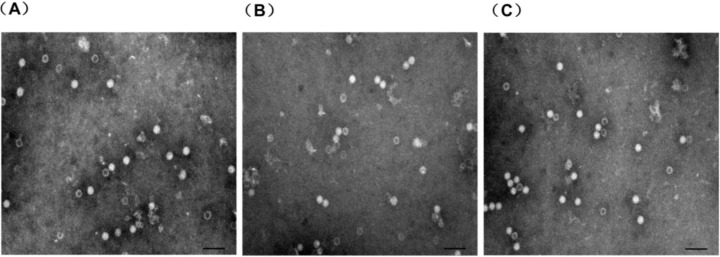
Analysis of chimeric Cap particles by electron microscopy. Electron microscopy of negatively stained purified chimeric Cap particles (**A**) recombinant protein Cap-T (**B**) recombinant protein Cap-B (**C**) recombinant protein Cap-TB. Scale bar indicates 100 nm.

The proteins were examined using electron microscopy analysis. The results indicated that the recombinant proteins could form VLPs with diameter of 17–25 nm (Figure 4), which was morphologically similar to those of the immature PCV2 virions.

### 3.3. PCV2-Specific Humoral Immune Responses

**Figure 5:**
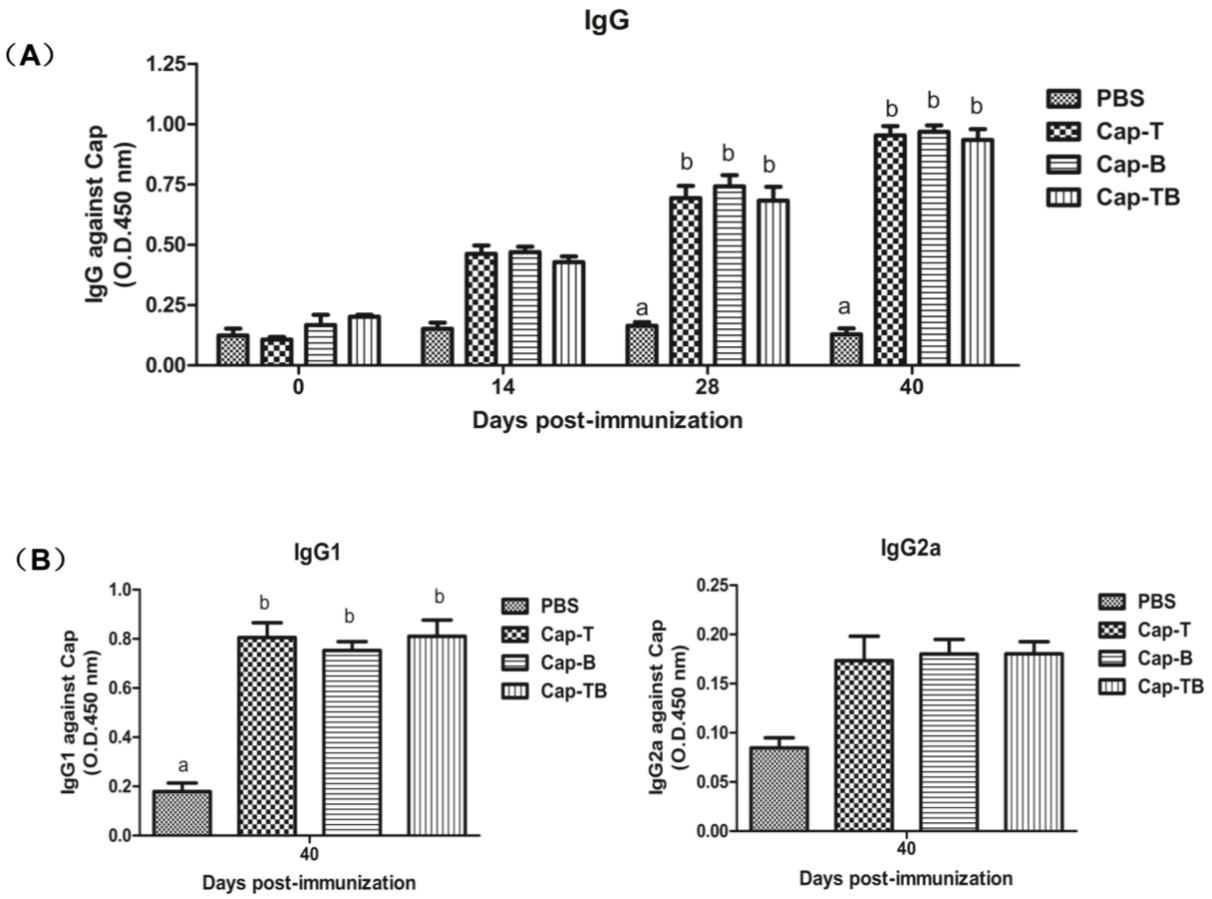
Cap-specific antibody responses in mice detected by indirect ELISA. (**A**) IgGs specific to Cap were found in serum samples at different times by indirect ELISA; (**B**) IgGs isotypes specific to Cap were detected by indirect ELISA on the day 40 post primary immunization. Data represent the mean ± SEM. Different letters (a,b) indicate a statistically significant difference between the different experimental groups (*p* < 0.05).

As shown in Figure 5A, the Cap protein-specific antibody responses could be measured in all mice immunized with Cap-T, Cap-B or Cap-Tb at 14 dpi (Figure 5). The antibody levels increased rapidly and reached a peak after the mice were booster-immunized at 40 dpi. The levels of Cap-specific IgG antibody for the groups inoculated with Cap-T, Cap-B or Cap-Tb had the highest OD values at 40 dpi; their mean OD values (0.953, 0.968 and 0.934, respectively) had significantly higher ELISA antibody titers than those of the mice vaccinated with PBS (*p* < 0.01).

IgG isotyping was determined by indirect ELISA at 40 dpi in order to analyze the immune response profile. The Cap-specific IgG1 and IgG2a levels were not detected at 0, 14, 28 dpi as previously described [27]. As shown in Figure 5B, the mice in all groups immunized with Cap-T, Cap-B or Cap-TB developed Cap-specific IgG1 and IgG2a. However, the IgG1 levels had significantly higher mean OD values than the IgG2a levels. Thus, these results suggested that Th2-dominant cellular immune response was induced in mice.

**Figure 6:**
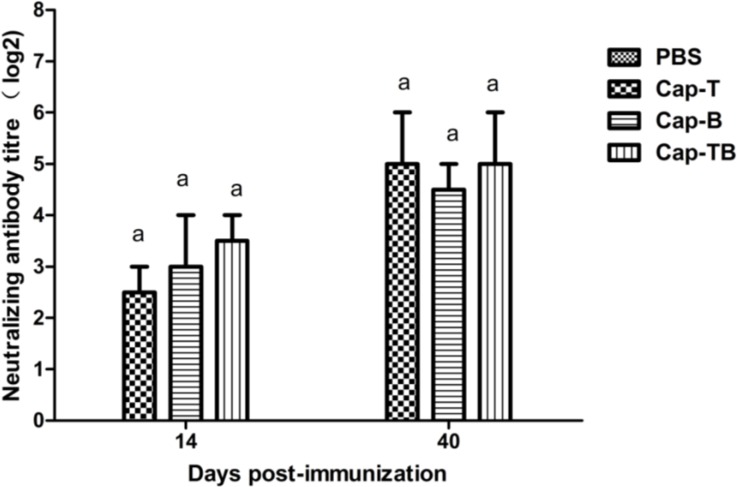
Detection of PCV2-specific neutralizing antibodies in sera. Neutralizing antibodies against PCV2 WH strain were measured in serum samples at day 14 and 40 dpi. The neutralizing antibody titers were calculated and expressed as the log2 of the reciprocal of the highest serum dilution that was able to completely block PCV2-infection in PK-15 cells. Data represent the mean ± SEM. Different letters (a,b) indicate a statistically significant difference between the different experimental groups (*p* < 0.05).

As shown in Figure 6, the mice immunized with Cap-T, Cap-B or Cap-TB developed mean the neutralization antibody titers of 2.5, 3 and 3.5 at 14 dpi, respectively; these titers increased to 5, 4.5 and 5 at 40 dpi, respectively. The results showed that the neutralizing antibody titers were not significantly different among the groups. The sera from the mice immunized with PBS did not exhibit any neutralizing antibody throughout the experiment.

### 3.4. CSFV Peptide-Specific Immune Response

**Figure 7:**
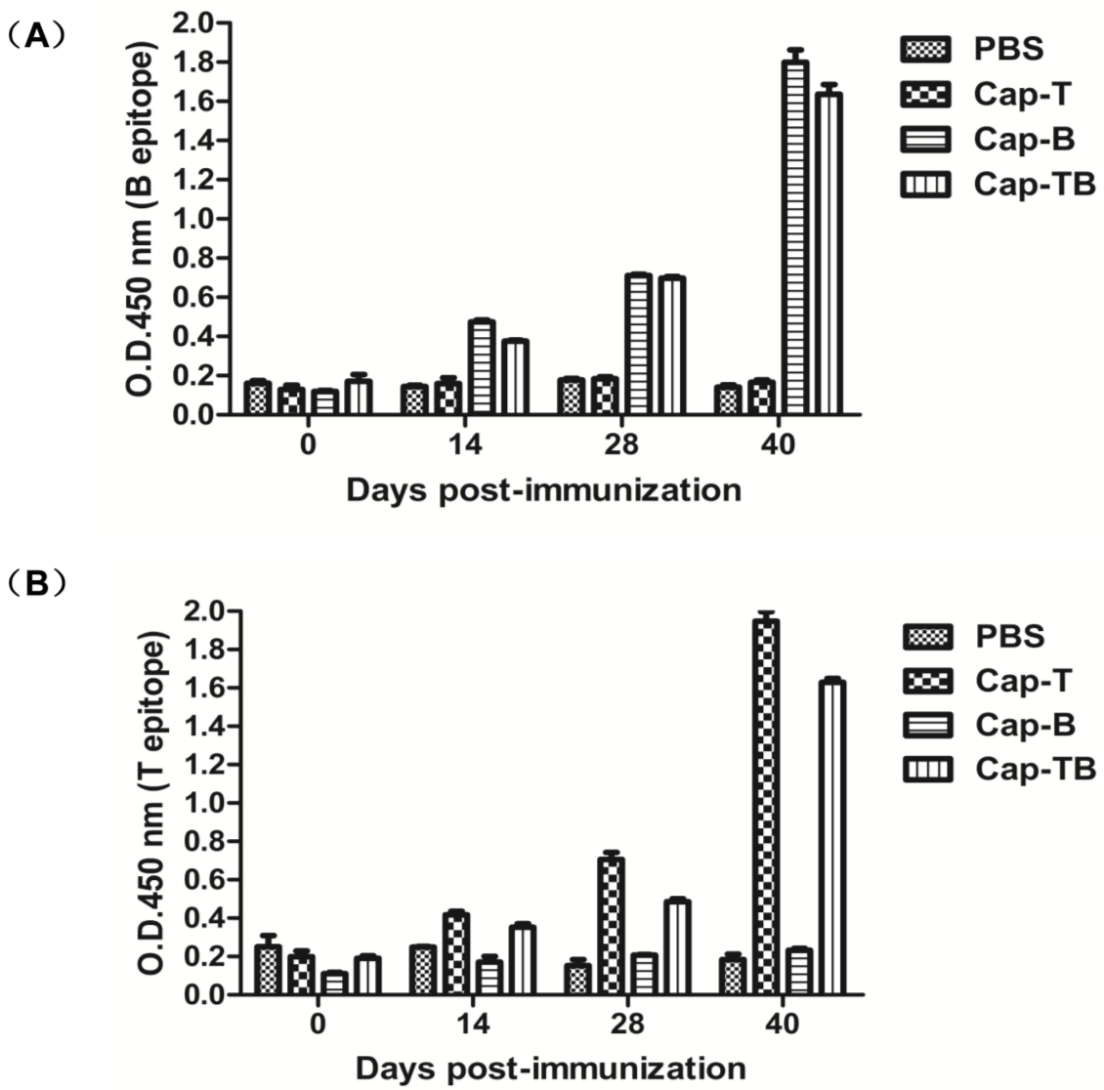
Specific antibody responses against CSFV T-epitope and B-epitope. (**A**) IgGs specific antibodies against B-epitope (693–716 aa) were detected at different times by indirect ELISA; (**B**) IgGs specific antibodies against T-epitope (1446–1460 aa) were detected at different times by indirect ELISA. Data represent the mean ± SEM.

To analyze whether Cap-T, Cap-B or Cap-TB could induce CSFV peptide-specific immune responses after immunization, we determined the T-epitope-specific antibodies in the immunized mice through indirect ELISA. As shown in Figure 7A, the T-peptide-specific antibodies were not detected in the sera of PBS and Cap-B. By contrast, all animals immunized with Cap-T and Cap-TB were positive at 28 dpi. The mean titers of T-epitope-specific antibodies significantly increased in the mice immunized with Cap-T and Cap-TB and were significantly higher than those in the mice vaccinated with PBS and Cap-B (*p* < 0.01).

Moreover, the B-epitope-specific antibodies in the immunized mice were measured with an indirect ELISA. As shown in Figure 7B, the antibody titer reached a detectable level in the vaccinated Cap-B and Cap-TB groups compared to the group immunized with PBS and Cap-T at 28 dpi. The mean titers of B-epitope-specific antibodies significantly increased in the mice immunized with Cap-B and Cap-TB were significantly higher than those in the mice vaccinated with PBS and Cap-T at 40 dpi. This difference was statistically significant (*p* < 0.01).

### 3.5. Cellular Immune Response

**Figure 8:**
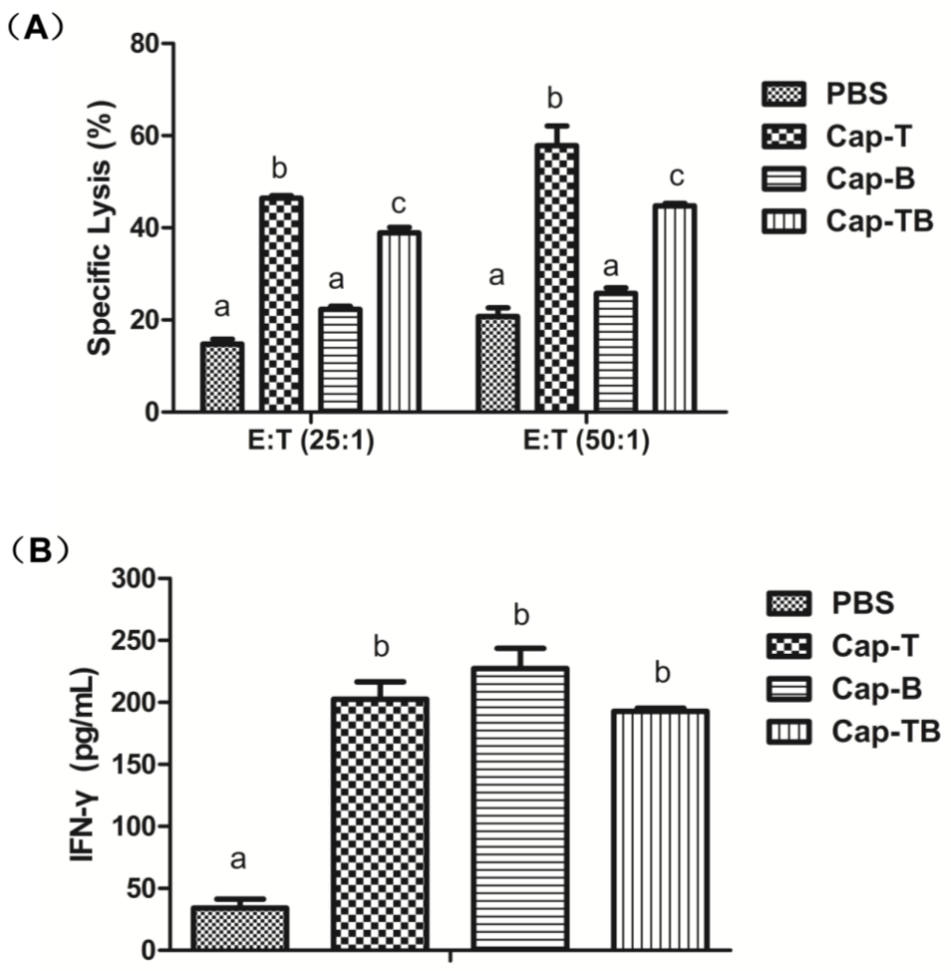
Specific anti-CSFV T-epitope CTL response and IFN-γ responses generated in mice. (**A**) Average of specific lysis per group of mice. The CTL activity was assayed by LDH release assay; (**B**) The levels of IFN-γ per group in serum samples of mice. Data represent the mean ± SEM. Different letters (a,b) indicate a statistically significant difference between the different experimental groups (*p* < 0.05).

To confirm whether the CTL activity of the recombinant proteins immunized in mice, we performed an LDH release assay. The results of specific cytotoxic activities are shown in Figure 8A. The mean CTL activity in the mice immunized with Cap-T and Cap-TB was 46.46% and 38.92%, respectively, at an E:T ratio of 50:1. The mean CTL activity was significantly higher in the mice immunized with Cap-B than that in the mice induced with Cap-B (*p* < 0.05). Compared with the control group, the specific CTL activity with the highest specific lysis reached 57.83% and 44.76% at an E:T ratio of 25:1 in the Cap-T and Cap-TB groups, respectively. Thus, CTL activity could be induced in the mice immunized with Cap-T and Cap-TB.

To characterize the cell-mediated immune responses in the mice immunized with VLPs, we determined the interferon γ (IFN-γ) production in the serum through ELISA. As shown in Figure 8B, the mean IFN-γ levels of 202.57, 227.33 and 192.79 pg/mL were detected in the mice inoculated with Cap-T, Cap-B and Cap-TB, respectively. The mean IFN-γ levels were not significantly different among the three groups (*p* > 0.05). By contrast, the mean IFN-γ level (34.19 pg/mL) of the sera from the mice immunized with PBS was lower than those of the other groups.

## 4. Discussion

VLPs did not contain viral genome can structurally mimic the authentic virus and have immunogenic potentials to regulate dendritic cells or other antigen-presenting cells [20,27,28]. Previous studies have shown that VLPs can be generated through genetic engineering, and could be used as a platform for gene or drug delivery and vaccine design [20,23]. VLPs of animal viruses have been used as platforms to deliver the foreign epitopes and induce immune responses against exogenous antigens [20,29].

Baculovirus expression system has been extensively applied into research and production of subunit vaccines, particularly for VLPs [30]. PPV VLPs is an antigen delivery system; the foreign gene CD8^+^ T-cell epitope from the LCMV nucleoprotein has been successfully delivered by PPV VLPs in the baculovirus expression system [29]. These results confirmed that the PPV VLPs of chimeras stimulate a potent CTL response and can protect mice against the lethal LCMV infection [24]. Many studies showed that PPV VLPs is a safe and non-replicating antigen carrier for developing chimeric VLP vaccines [31].

Lan-Jun Liu *et al.* (2008) reported that PCV2 capsid protein expressed in Tn5 cells could self-assemble into VLPs and release into the culture medium [32]. The *N*-terminus (1–39 aa) of the Cap protein contains NLS, which is unnecessary for forming VLPs [16]. Huiying Fan *et al.* (2008) reported that the pseudotyped baculovirus with vesicular stomatitis virus glycoprotein (VSV-G) was used as a vector expressing the capsid protein of PCV2 to develop a new generation of vaccines against PCV2 infection [33]. However, all of the above research did not confirm that PCV2 VLPs could be used as antigen vehicle carrying foreign epitopes. In this study, we evaluated the potential of PCV2 VLPs as a vessel for carrying foreign epitopes. Three Cap recombinants (Cap-T, Cap-B and Cap-TB) containing the CD8^+^ T-cell epitope or B-cell epitope from CSFV were produced. Cap-T, Cap-B and Cap-TB proteins were expressed in the baculovirus system as confirmed by Western blotting and IFA (Figure 2 and Figure 3). The electron microscopy results showed that all recombinant proteins could form VLPs 17–25 nm in diameter (Figure 4). Thus, replacing the NLS residues of the PCV2 Cap protein with foreign epitope not affect the formation of VLPs.

The results showed these recombinant proteins could induce high levels of the Cap-specific IgG antibody compared with the negative control (Figure 5A). However, the antibody levels were not significantly different among the Cap-T, Cap-B and Cap-TB groups. The high levels of the Cap-specific IgG1 antibody were similar among the groups but were not detected in the negative control (Figure 5B). By contrast, these recombinant proteins did not induce high levels of the Cap-specific IgG antibody compared with the negative control. IgG1 antibody is characteristic of the Th2 response. Thus, Cap-T, Cap-B and Cap-TB proteins can activate the Th2 response. The PCV2-specific neutralizing antibody was evaluated at 40 dpi. The negative group did not exhibit neutralization activity throughout the experiment. Moreover, the Cap-T, Cap-B and Cap-TB groups had similar PCV2-specific neutralizing antibodies (Figure 6). These results were consistent with the finding that the mice immunized with Cap-T, Cap-B and Cap-TB produced significant levels of Cap-specific IgG. In addition, all recombinant proteins (Cap-T, Cap-B and Cap-TB) could induce T-epitope-specific IgG or B-epitope-specific IgG (Figure 7). However, no neutralization activity was detected against CSFV in any groups, even in the mice immunized with Cap-B or Cap-TB, which carried the B epitope from CSFV. Therefore, the *N*-terminus of Cap without the NLS sequence is unsuitable for inserting foreign B-cell epitopes.

The amounts of IFN-γ in the groups vaccinated with Cap-T, Cap-B and Cap-TB were significantly higher than that in the control group (Figure 8B). However, the mean IFN-γ response was not significantly different in the Cap-T, Cap-B and Cap-TB groups. Moreover, the specific CTL response was detected in the Cap-T and Cap-TB groups. Our results showed that the specific CTL killing with the highest specific lysis reached 57.83% and 44.76% at an E: T ratio of 25:1 in the Cap-T and Cap-TB groups, respectively (Figure 8A). Thus, the Cap-T and Cap-TB protein could induce specific CTL responses in the immunized mice. Our data confirmed that the *N*-terminus of Cap without NLS sequence is suitable for inserting foreign T-cell epitopes.

In conclusion, our results demonstrated that replacing and inserting foreign epitopes at the *N*-terminus of Cap did not affect the structure of the protein and its ability to form VLPs. The mice immunized with the Cap-T, Cap-B and Cap-TB proteins produced Cap-specific immunoreaction. Moreover, specific CTL response was detected in the Cap-T and Cap-TB groups. Therefore, the *N*-terminus of Cap without NLS sequence could be the optimal position for carrying foreign T-cell epitopes.
